# ^15^N, ^13^C and ^1^H resonance assignments of FKBP12 proteins from the pathogenic fungi *Mucor circinelloides* and *Aspergillus fumigatus*

**DOI:** 10.1007/s12104-019-09878-x

**Published:** 2019-02-01

**Authors:** Sophie M. C. Gobeil, Benjamin G. Bobay, Leonard D. Spicer, Ronald A. Venters

**Affiliations:** 1 Department of Biochemistry, Duke University, Durham, NC USA; 2 Duke University NMR Center, Duke University, Durham, NC USA; 3 Department of Radiology, Duke University, Durham, NC USA

**Keywords:** FK506, FKBP12, Calcineurin, *Mucor circinelloides*, *Aspergillus fumigatus*

## Abstract

Invasive fungal infections are a leading cause of death in immunocompromised patients and remain difficult to treat since fungal pathogens, like mammals, are eukaryotes and share many orthologous proteins. As a result, current antifungal drugs have limited clinical value, are sometimes toxic, can adversely affect human reaction pathways and are increasingly ineffective due to emerging resistance. One potential antifungal drug, FK506, establishes a ternary complex between the phosphatase, calcineurin, and the 12-kDa peptidyl-prolyl isomerase FK506-binding protein, FKBP12. It has been well established that calcineurin, highly conserved from yeast to mammals, is necessary for invasive fungal disease and is inhibited when in complex with FK506/FKBP12. Unfortunately, FK506 is also immunosuppressive in humans, precluding its usage as an antifungal drug, especially in immunocompromised patients. Whereas the homology between human and fungal calcineurin proteins is > 80%, the human and fungal FKBP12s share 48–58% sequence identity, making them more amenable candidates for drug targeting efforts. Here we report the backbone and sidechain NMR assignments of recombinant FKBP12 proteins from the pathogenic fungi *Mucor circinelloides* and *Aspergillus fumigatus* in the apo form and compare these to the backbone assignments of the FK506 bound form. In addition, we report the backbone assignments of the apo and FK506 bound forms of the *Homo sapiens* FKBP12 protein for evaluation against the fungal forms. These data are the first steps towards defining, at a residue specific level, the impacts of FK506 binding to fungal and mammalian FKBP12 proteins. Our data highlight differences between the human and fungal FKBP12s that could lead to the design of more selective anti-fungal drugs.

## Biological context

**Fig. 1:**
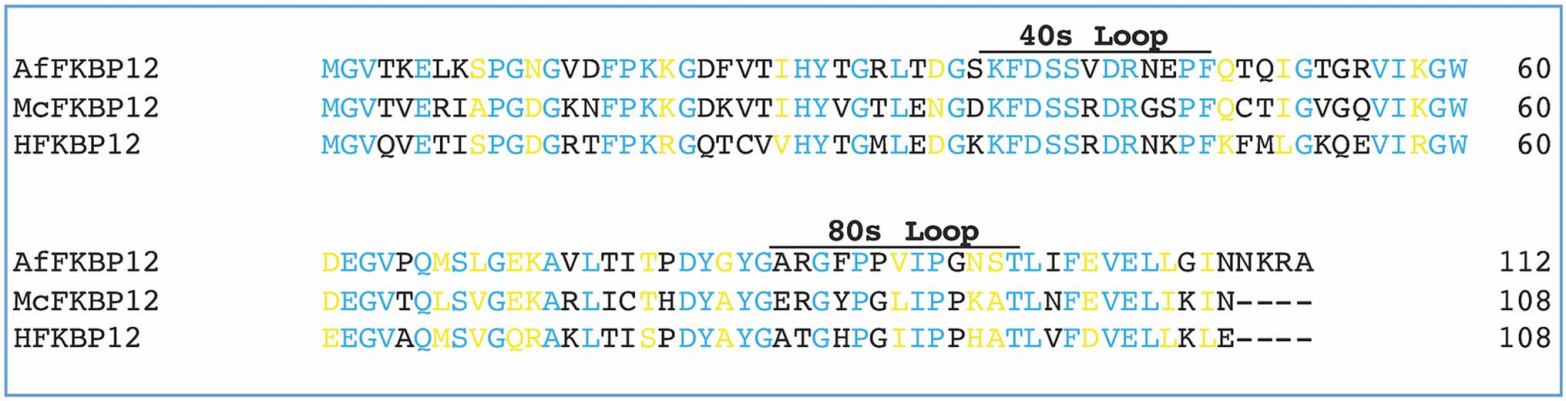
Alignment of FKBP12 sequences. Sequence alignment of the FKBP12s from *M. circinelloides*, *A. fumigatus*, and *H. sapiens*. Identical (cyan) and highly conserved residues (yellow), and different residues (black) are noted. In *H. sapiens* FKBP12, the residues making up the 40s and 80s loops are known to form a surface for the interaction with calcineurin

FKBP12s are monomeric ~ 12 kDa *cis–trans* peptidyl-prolyl isomerases that play key roles in homeostasis both in invasive pathogenic fungi and humans. FKBP12s have been shown to bind the macrolide FK506, currently used clinically as an immunosuppressive drug preventing graft rejection. The FK506/FKBP12 complex subsequently binds to and inhibits the Ca^2+^/calmodulin-dependent protein phosphatase calcineurin (CaN). In humans, this, in turn, inhibits the downstream nuclear factor of activated T-cells (NF-AT) which is implicated in interleukin-2 (IL-2) transcription and T-cell activation; while in fungi it inhibits the nuclear translocation of the transcription factor *Crz1p*/*Tcn1p* regulating the expression of genes involved in cell wall integrity, growth and drug resistance (Aramburu et al. 2001; Hogan et al. 2003). It has been demonstrated that CaN is required for virulence of the pathogenic fungi *Candida albicans, Candida glabrata, Mucor circinelloides*, and *Aspergillus fumigatus* thus defining both FKBP12 and CaN as potential broad-spectrum anti-fungal drug targets (Juvvadi et al. 2014, 2017). Although FK506 is active in vitro against the major invasive fungal pathogens, the conservation of the CaN pathway between fungi and mammals hamper its therapeutic efficacy as an antifungal as it is immunosuppressive for the human host. Since the homologies between the human and fungal FKBP12 proteins are low (48–58%) when compared to the homologies between the human and fungal calcineurin proteins (> 80%), targeting the selective inhibition of fungal FKBP12s is a promising drug discovery strategy (Fig. 1).

Our hypothesis is that by combining solution-state NMR, X-ray crystallography, and molecular dynamics simulation to identify differences in FKBP12 binding to FK506, we will obtain important insights to overcome the fungal versus human specificity obstacle. Employing this approach, combined with structure-informed site-directed mutagenesis and in vivo, in vitro, and in silico studies, we are endeavoring to define novel targetable fungal-specific areas in the calcineurin complex that are critical for fungal pathogenesis but do not impede the host immune system (Juvvadi et al. 2017). Here we describe the NMR resonance assignments of fungal FKBP12s from *M. circinelloides* and *A. fumigatus*, in both the apo and FK506 bound forms, as an initial and necessary step towards this goal. Assignment of *H. sapiens* FKBP12 was reported previously (Sapienza et al. 2011; Mustafi et al. 2013) and was repeated here for comparison purposes. Using these solution-state NMR data, we will elucidate, at a residue specific level, the impact of FK506 binding to both human and fungal FKPB12 proteins providing important insights towards overcoming the specificity obstacle.

## Methods and experiments

### DNA constructs, expression and purification

*Mucor circinelloides, A. fumigatus* and *H. sapiens* FKBP12 constructs were obtained from GenScript (Piscataway, NJ) in the pET-15b vector. The plasmids, containing the proteins with a His6-tag at the N-terminus and a thrombin cleavage site, were transformed into *E. coli* BL21(DE3) cells and plated on LB-agar containing ampicillin (100 µg/mL). For NMR, uniformly [^15^N]- and [^13^C, ^15^N]-labeled proteins were overexpressed at 25 °C in modified M9 minimal medium containing 1 g/L ^15^NH_4_Cl and 2 g/L (*A. fumigatus* and *H. sapiens)* or 4 g/L (*M. circinelloides*) ^13^C-glucose (Cambridge Isotopes, Tewksbury, MA). Cells were propagated to an OD_600_ of 0.6 and induced with 1 mM Isopropyl β-d-1-thiogalactopyranoside (IPTG) for 4 h (*A. fumigatus* and *H. sapiens*) or 16 h (*M. circinelloides*). Cells were then harvested by centrifugation at 4 °C for 15 min at 6000×*g* and the pellet stored at − 20 °C until purification.

The frozen pellets were resuspended in 30 mL of lysis buffer (50 mM sodium phosphate, 500 mM NaCl, pH 8.0) supplemented with 1 mL of protease inhibitor cocktail (Sigma, St. Louis, MO) and 1 mM phenylmethane sulfonyl fluoride (PMSF). Lysis was performed using a French press or three cycles of 30 s of sonication at a power of 12 watts with a 2-min rest interval on ice. Lysate was clarified by centrifugation (4 °C, 15 min at 20,000×*g*) and supernatant was loaded onto a Ni–NTA column. Protein was eluted using a 0–1 M gradient of imidazole. Fractions containing FKBP12 protein were identified using SDS-PAGE revealed by the Coomasie Blue staining method and combined to be dialyzed four times for one hour each into 50 mM sodium phosphate, 500 mM NaCl, pH 8.0 buffer to remove the imidazole. The 6X-His tag was then cleaved for 16 h at 4 °C using 1U of thrombin per 100 µg of total protein (GE Healthcare). The cleaved proteins were then loaded onto the Ni–NTA column to remove the cleaved 6X-His tag and any potentially uncleaved protein. Subsequent size-exclusion chromatography using a Sephacyrl S100HR XK26/60 FPLC column yielded pure protein. Typical yields were 20 mg/L of > 98% pure protein as determined by SDS-PAGE gels stained with Coomasie Blue.

### Solution NMR spectroscopy

[^13^C,^15^N]-labeled and [^15^N]-labeled samples were concentrated to approximately 0.4–0.7 mM and buffer exchanged into NMR buffer (20 mM sodium phosphate, 100 mM NaCl, 0.02% NaN3 and 5% D_2_O at pH 6.0) using a 3000 MWCO Amicon concentrator. All NMR experiments were performed at 25 °C, as calibrated with a standard methanol sample. NMR backbone resonance assignments for the FKBP12 proteins (apo and FK506 bound) were determined using the non-uniform sampling NMR experiments (Venters et al. 2005; Coggins and Zhou 2008): HNCO, HN(CA)CO, HNCA, HN(CO)CA, HN(CA)CB, HN(COCA)CB, HA(CA)NH, and HA(CACO)NH. Sidechain assignments for the apo forms of *M. circinelloides* and *A. fumigatus* were determined using this same sample and the 4D HCC(CO)NH experiment in addition to the HCCH TOCSY experiment (Coggins and Zhou 2008) on a sample exchanged into the same NMR buffer containing 100% D_2_O. All NMR experiments were performed on a Bruker Avance 14.1T (600 MHz) spectrometer equipped with a triple-resonance cryoprobe and pulsed-field Z-gradient. NMR data were processed using NMRPipe (Delaglio et al. 1995) and analyzed using Sparky (Goddard and Kneller 2006) and NMRViewJ version 8.0 (Johnson and Blevins 1994). Chemical shifts were referenced to an external 2,2-dimethyl-2-silapentane-5-sulfonate (DSS) sample. The programs PINE (Lee and Markley 2018), AutoAssign (Zimmerman et al. 1997) and RunAbout in NMRViewJ (Johnson and Blevins 1994) were used to determine the initial chemical shift assignments with discrepancies or missing spin systems determined manually. For comparison purposes, the backbone resonance assignments of the *H. sapiens* form of the protein with and without bound FK506 have also been determined using the same buffer and methods. For all proteins studied here, the FK506 bound assignments were determined de novo and did not rely on the assignments determined for the apo versions of the proteins.

### Assignments and data deposition

**Fig. 2:**
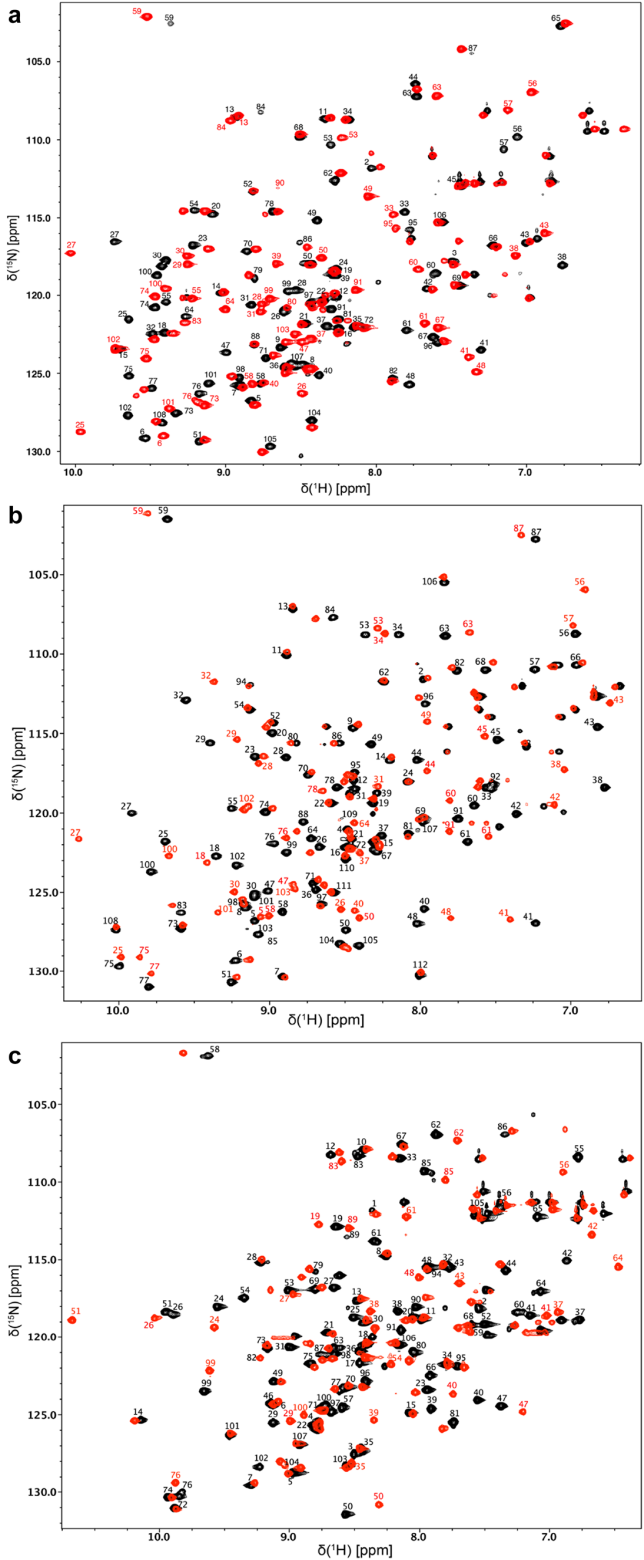
Two-dimensional ^1^H–^15^N HSQC spectra. **a***M. circinelloides*, **b***A. fumigatus*, and **c***H. sapiens* FKBP1*2* in the apo and FK506 bound forms. Assigned peaks for the apo (black) protein are labeled. Only select peaks in the FK506 bound (red) spectrum are labeled to indicate crosspeaks with significant binding shifts or for assignment clarity

**Fig. 3:**
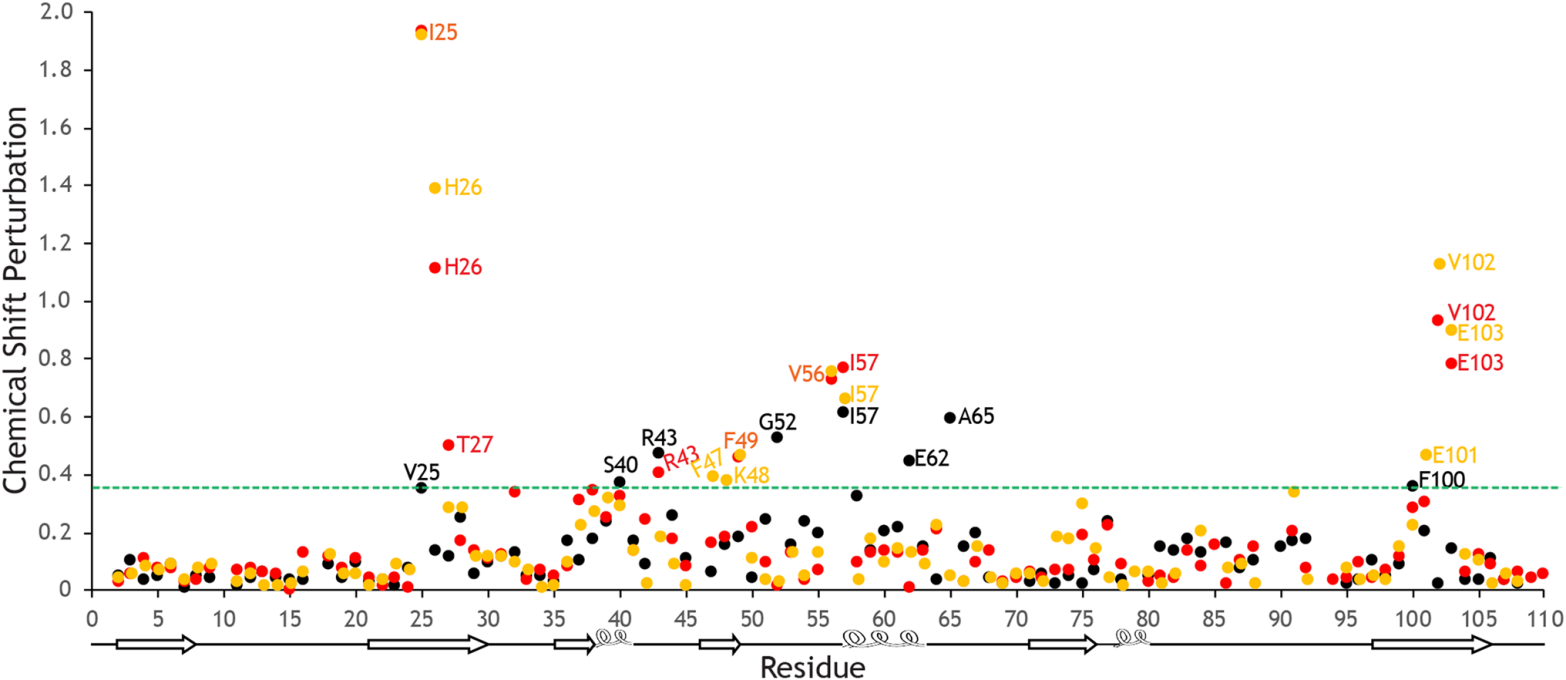
Chemical shift perturbations for FK506 binding. The chemical shift perturbations calculated (Williamson 2013) for *M. circinelloides* (orange), *A. fumigatus* (red), and *H. sapiens* (black) FKBP12 upon binding of FK506. The scaling factor, α, for the ^15^N shifts is set to 0.14 relative to the ^1^H shifts as suggested by Williamson (2013). The shifts observed for residues in the FKBP12 proteins from the pathogenic fungi are very similar, whereas clear differences are seen compared to the human protein. The green dashed line represents chemical shift perturbations at least one standard deviation above the overall average

The monomeric ~ 12 kDa *M. circinelloides* (108 amino acid) and *A. fumigatus* (112 amino acid) FKBP12 proteins share 65% sequence identity between themselves and ~ 57% sequence identity to the *H. sapiens* protein (108 amino acid) (Fig. 1). Much like the previously reported assignment of the *H. sapiens* FKBP12 protein (Sapienza et al. 2011; Mustafi et al. 2013), the two fungal FKBP12 proteins gave rise to well dispersed and high quality ^1^H–^15^N HSQC spectra (Fig. 2). Each of the ^1^H–^15^N HSQC figures presents the apo (black) form and the FK506 (red) bound form of the proteins. Only selected residues with large chemical shift perturbations or those requiring clarification are labeled in the FK506 bound HSQC spectra. Figure 3 presents the chemical shift perturbations (Williamson 2013) for each of the residues from each protein upon binding to FK506.

The final level of completeness obtained for the backbone resonance assignments were 96–99% for the non-proline backbone ^1^H_N_ and ^15^N resonances, 95–99% for ^13^CO, 98–99% for ^13^C_α_, 92–99% for ^13^C_β_ and 94–98% for ^1^H_α_ (Table 1). 96% of the expected sidechain resonances were assigned for apo *A. fumigatus* FKBP12 including the aromatic ring resonances. Apo *M. circinelloides* FKBP12, on the other hand, is missing the most assignments most likely due to line broadening caused by protein dynamics; because of this, only 65% of the expected sidechain resonances were assigned for this protein. FK506 binding appears to stabilize the *M. circinelloides* FKBP12 thus facilitating the assignment. The assignment of the *A. fumigatus* FKBP12 protein was found to be the most complete.

**Table 1:** Percent assigned

Protein	Form	^1^H_N_/^15^N	^13^CO	^13^C_α_	^13^C_β_	^1^H_α_	^1^H_β_
*M. circinelloides*	apo	96.1% (98/102)	95.4% (103/108)	98.1% (106/108)	92.5% (86/93)	94.4% (102/108)	80.6% (75/93)
	FK506	98.0% (100/102)	99.1% (107/108)	99.1% (107/108)	98.9% (92/93)	98.1% (106/108)	
*A. fumigatus*	apo	99.0% (103/104)	99.1% (111/112)	99.1% (111/112)	99.0% (95/96)	98.2% (110/112)	99.0% (95/96)
	FK506	99.0% (103/104)	99.1% (111/112)	99.1% (111/112)	99.0% (95/96)	97.3% (109/112)	
*H. sapiens*	apo	98.0% (99/101)	99.1% (107/108)	99.1% (107/108)	98.9% (94/95)	96.3% (104/108)	
	FK506	99.0% (100/101)	98.1% (106/108)	98.1% (106/108)	96.8% (92/95)	94.4% (102/108)	

The ^1^H, ^15^N and ^13^C resonance assignments for apo and FK506 bound *M. circinelloides, A. fumigatus* and *H. sapiens* FKBP12 proteins have been deposited in the BioMagResBank (http://www.bmrb.wisc.edu) under the accession codes 27734, 27737, 27732, 27733, 27738 and 27739 respectively.

The chemical shift assignments presented here are the first step towards discerning differences between the human and fungal FKBP12 proteins in their response to ligand binding. It is clear from the calculated chemical shift perturbations (Williamson 2013) that residues in the FKBP12 proteins from the two pathogenic fungi respond similarly to the binding of the inhibitory drug FK506 (Fig. 3). However, this response is significantly different from the human protein. The fungal proteins both exhibit chemical shift perturbations at least one standard deviation above the overall average (0.39 for all three proteins) for residues Ile25, His26, Phe49, Val56, Ile57, Val102 and Glu103. These residues are conserved between these two fungal proteins. Additionally, *M. circinelloides* residues Phe47, Lys48 and Glu101 and *A. fumigatus* residues Thr27 and Arg43 exceed this chemical shift threshold. In *H. sapiens* FKBP12, these residues are also conserved with the exception of Ile25, which is a Val, but the shifts observed are much less significant. In contrast, the residues most affected by FK506 binding in the human protein are Val25, Ser40, Arg43, Gly52, Ile57, Glu62, Ala65, and Phe100 indicating a possible different binding orientation.

We postulate that our assignments and chemical shift data, when combined with structures and the investigation of the dynamic behavior of these proteins will reveal human and fungal specific differences that can be exploited to design new anti-fungal drugs that do not concomitantly suppress the human immune system.

## Conclusion

^1^H, ^15^N, and ^13^C backbone and sidechain resonance assignments for the apo and the backbone assignments for the FK506 bound forms of FKBP12 proteins from the pathogenic fungi *M. circinelloides* and *A. fumigatus* were achieved using double- and triple-resonance non-uniform sampling NMR experiments. We also present our backbone assignments for the human form of this protein in both the apo and FK506 bound states and compare them to previously published results. These assignments provide the means to investigate differences in ligand binding and will be used to investigate the dynamics of these proteins both in the apo and ligand bound state providing insights into the structure/function/dynamics relationships and in defining differences that could be exploited in the design of future anti-fungal drugs that are not immunosuppressive to the human host.
